# Measuring myocardial extracellular volume of the right ventricle in patients with congenital heart disease

**DOI:** 10.1038/s41598-021-81440-z

**Published:** 2021-01-29

**Authors:** Nadya Al-Wakeel-Marquard, Tiago Ferreira da Silva, Sarah Jeuthe, Sanaz Rastin, Frédéric Muench, Darach O h-Ici, Sevim Yilmaz, Felix Berger, Titus Kuehne, Daniel R. Messroghli

**Affiliations:** 1 Department of Congenital Heart Disease - Pediatric Cardiology, German Heart Center Berlin, Berlin, Germany; 2 Institute for Imaging Science and Computational Modelling in Cardiovascular Medicine, Charité – Universitätsmedizin Berlin, corporate member of Freie Universität Berlin, Humboldt-Universität zu Berlin, and Berlin Institute of Health, Berlin, Germany; 3 DZHK (German Centre for Cardiovascular Research), partner site Berlin, Berlin, Germany; 4 Department of Internal Medicine - Cardiology, German Heart Center Berlin, Berlin, Germany; 5 Max-Delbrück-Center for Molecular Medicine, Berlin, Germany; 6 Centre for Paediatric and Adolescent Medicine, St. Joseph´S Hospital, Berlin, Germany; 7 Department of Internal Medicine - Cardiology and Conservative Intensive Care, Vivantes Klinikum Im Friedrichshain, Berlin, Germany; 8 Division of Cardiology, Department of Pediatrics, Charité – Universitätsmedizin Berlin, corporate member of Freie Universität Berlin, Humboldt-Universität zu Berlin, and Berlin Institute of Health, Berlin, Germany; 9 Department of Internal Medicine and Cardiology, Charité – Universitätsmedizin Berlin, corporate member of Freie Universität Berlin, Humboldt-Universität zu Berlin, and Berlin Institute of Health, Berlin, Germany

**Keywords:** Cardiology, Medical research

## Abstract

The right ventricle´s (RV) characteristics—thin walls and trabeculation—make it challenging to evaluate extracellular volume (ECV). We aimed to assess the feasibility of RV ECV measurements in congenital heart disease (CHD), and to introduce a novel ECV analysis tool. Patients (n = 39) and healthy controls (n = 17) underwent cardiovascular magnetic resonance T1 mapping in midventricular short axis (SAX) and transverse orientation (TRANS). Regions of interest (ROIs) were evaluated with regard to image quality and maximum RV wall thickness per ROI in pixels. ECV from plane ROIs was compared with values obtained with a custom-made tool that derives the mean T1 values from a “line of interest” (LOI) centered in the RV wall. In CHD, average image quality was good (no artifacts in the RV, good contrast between blood/myocardium), and RV wall thickness was 1–2 pixels. RV ECV was not quantifiable in 4/39 patients due to insufficient contrast or wall thickness < 1 pixel. RV myocardium tended to be more clearly delineated in SAX than TRANS. ECV from ROIs and corresponding LOIs correlated strongly in both directions (SAX/TRANS: r = 0.97/0.87, *p* < 0.001, respectively). In conclusion, RV ECV can be assessed if image quality allows sufficient distinction between myocardium and blood, and RV wall thickness per ROI is ≥ 1 pixel. T1 maps in SAX are recommended for RV ECV analysis. LOI application simplifies RV ECV measurements.

## Introduction

Myocardial fibrosis contributes to the development of heart failure in adult congenital heart disease (CHD)^1^. Both focal^2–5^ and diffuse myocardial fibrosis^6–11^ have been described in different forms of CHD using cardiovascular magnetic resonance (CMR). This fibrosis is associated with decreased exercise capacity, ventricular dysfunction, and arrhythmia. CMR T1 mapping is a non-invasive tool to quantify diffuse myocardial fibrosis. The expansion of the interstitium as a marker for fibrosis can be assessed by measuring the myocardial volume of distribution of gadolinium-based contrast agents (myocardial extracellular volume, ECV)^12^.

While management of acquired heart diseases is focused on the left ventricle (LV), in CHD it is frequently the right ventricle (RV) that is affected by the underlying disease. Significantly elevated levels of myocardial LV ECV derived from CMR have been found in mainly right ventricular congenital pathologies as tetralogy of Fallot (TOF)^7,8^, yet data on myocardial ECV of the RV are limited^8,13^. The thin wall and distinct trabeculation of the RV complicate the differentiation between blood and myocardium required for accurate ECV determination. Conventional planimetric region of interest (ROI) analysis is regarded as the standard approach for ECV analysis in the LV^14^, but it is cumbersome and time-consuming for small, thin structures such as the RV. In these situations, a displacement of the outer border of the ROI by 1 pixel can already significantly affect the average value of the ROI, and thus the results of ECV calculations. We developed a tool that does not require contouring of the RV wall. Instead, the RV wall is directly targeted in its center as a “line of interest” (LOI), without the necessity for delineating between myocardial and blood pixels, and thus facilitating a quick and simple evaluation of myocardial RV ECV.

The present study aimed a) to evaluate the feasibility of CMR-based measurements of RV ECV with T1 mapping in patients with CHD, and b) to introduce the novel LOI approach in comparison with conventional planimetric ROI analysis.

## Methods

Consecutive patients ≥ 14 years of age with CHD referred for clinical CMR were prospectively recruited for the study. Exclusion criteria were significant renal impairment, chronic or acute infection up to four weeks prior to CMR, non-magnetic resonance imaging compatible devices, claustrophobia, and defective datasets or missing hematocrit values. The control group consisted of healthy young adults > 18 years with no previous history or symptoms of cardiovascular disease, no kidney dysfunction, normal 12-lead electrocardiogram and blood pressure, and no contraindications to perform CMR. Controls underwent CMR only for study purposes, additional examination of any other organ was not performed.

The study was approved by the institutional ethics committee (Charité – Universitätsmedizin Berlin) following the ethical guidelines of the 1964 Declaration of Helsinki and its later amendments. All participants and guardians of individuals < 18 years gave written informed consent to participate in the study.

After exclusion of two patients and two controls with defective datasets and missing hematocrit values, a total of 39 CHD patients and 17 controls were available for further analysis.

### CMR Protocol

Subjects were studied using a clinical whole-body 1.5 T MR imaging system (Philips Healthcare, Best, The Netherlands). T1 mapping was performed with an optimized ECG-gated single-shot modified Look-Locker inversion-recovery (MOLLI) sequence^15^. Sequence parameters were: MOLLI scheme 3b(3b)3b(3b)5b, repetition time 2.4 ms, echo time 1.2 ms; flip angle 35°, slice thickness 8.0 mm. Images were acquired in a single midventricular plane in SAX and transverse orientation (TRANS) before and 15 min after bolus application of gadoterate meglumine (Gd-DOTA, Dotarem, Guerbet) or gadopentetate dimeglumine (Gd-DTPA, Magnevist, Bayer; n = 1) at a dose of 0.1 mmol/kg^16^.

### Image analysis

The open-source image reconstruction tool MRmap, run with IDL Virtual Machine version 8.3 (Exelis, VA), was applied to generate T1 maps after manual correction for body motion. With the help of a grid structure superimposed onto the T1 source images to allow visual registration, images sorted by inversion time were separately displaced in x or y direction to correct for misregistration^17^. T1 maps generated from the corrected T1 images were stored as DICOM files, and T1 data analyzed with the open-source software OsiriX^18^.

**Figure 1:**
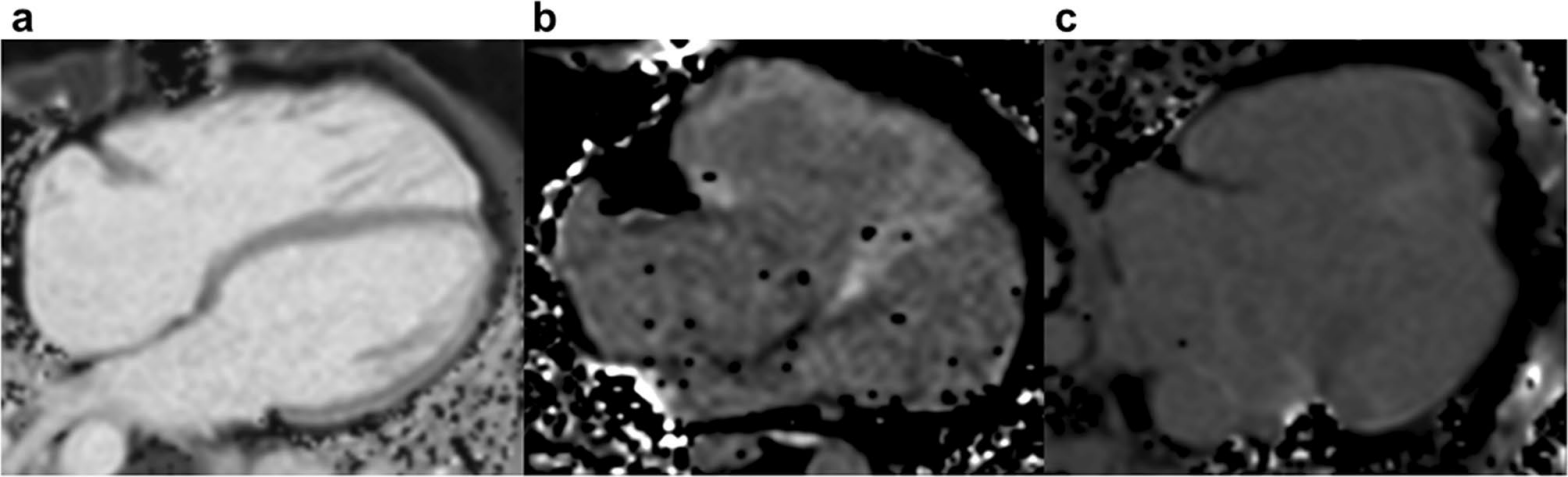
Grades of image quality. Native (panel **a**) and post-contrast T1 maps (panels **b** + **c**) with image quality rated as **a**: excellent (score grade 1) in a 39-year-old patient with repaired tetralogy of Fallot; **b**: sufficient (score grade 2) in a 37-year-old patient with d-transposition of the great arteries post atrial redirection with the Senning procedure; **c**: poor (score grade 3) in a 23-year-old patient with univentricular heart post Fontan palliation. Definitions of grades 1–3 are given in Table 1. Images were created using OsiriX Lite (v.12.0.0, https://www.osirix-viewer.com/).

**Figure 2:**
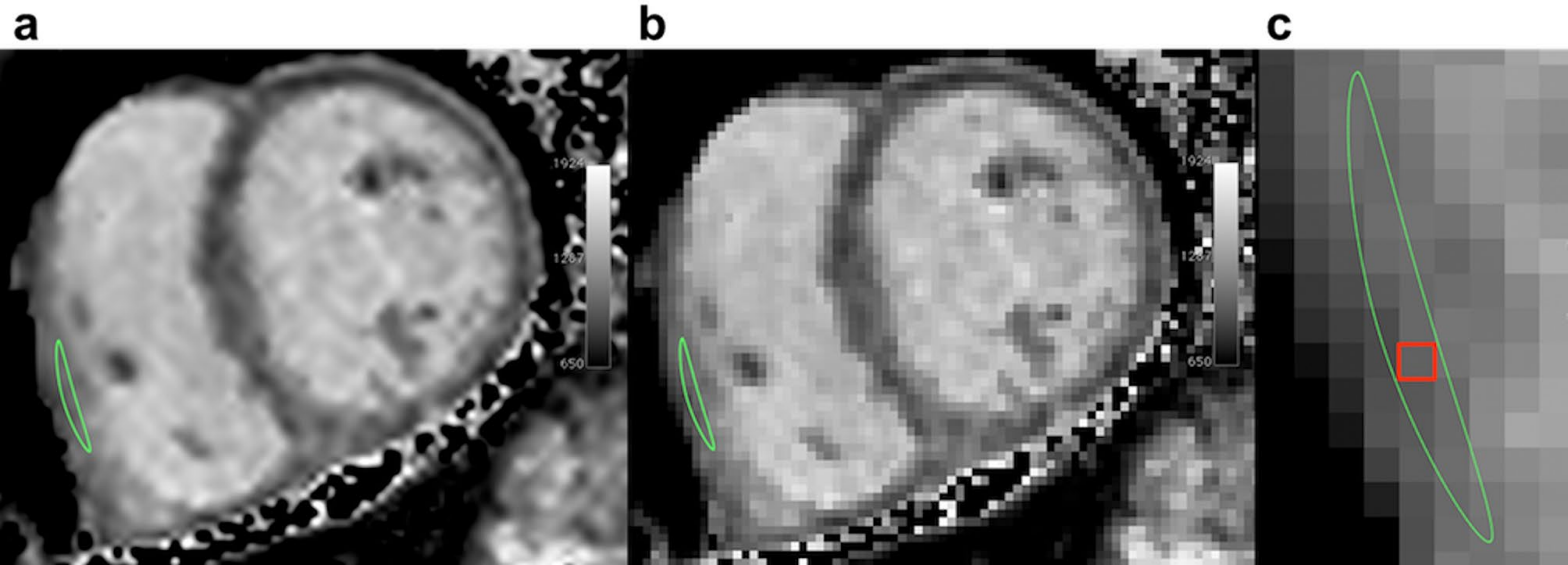
Image quality and wall thickness for T1 mapping of the right ventricle. Representative native T1 map of a 36-year-old patient with pulmonary stenosis demonstrating excellent image quality (score grade 1; panel **a**) and right ventricular wall thickness of 1 pixel per region of interest (score grade 2; panels **b** + **c**). Images were created using OsiriX Lite (v.8.5, https://www.osirix-viewer.com/).

Blood T1 was obtained from regions of interest (ROIs) delineated in the blood pool of the systemic (subaortic) ventricle. Myocardial ROIs with a minimum length of 10 mm were drawn in the LV wall (entire circumference for LV in subaortic location, and lateral wall for LV in subpulmonary location and in cases of univentricular repair) in SAX, and in the thickest, non-trabeculated part of the free, inferior or anterior wall of the RV in SAX and TRANS. ROIs were placed accurately in the myocardium to minimize partial voluming with adjacent blood or extramyocardial tissues. RV ROIs were evaluated with regard to image quality (Table 1; Fig. 1 and Fig. 2) and maximum RV wall thickness per ROI expressed in pixels (grade 1: > 2 pixels, grade 2: 1–2 pixels, grade 3: < 1 pixel; Fig. 2).

**Table 1:** Grading of image quality for evaluation of right ventricular regions of interest.

Grade	Definition
1	No artifacts in RV, good contrast between blood and myocardium
2	Minor artifacts in RV, good/sufficient contrast between blood and myocardium
3	Major artifacts in RV and/or insufficient contrast between blood and myocardium

RV = right ventricle.

**Figure 3:**
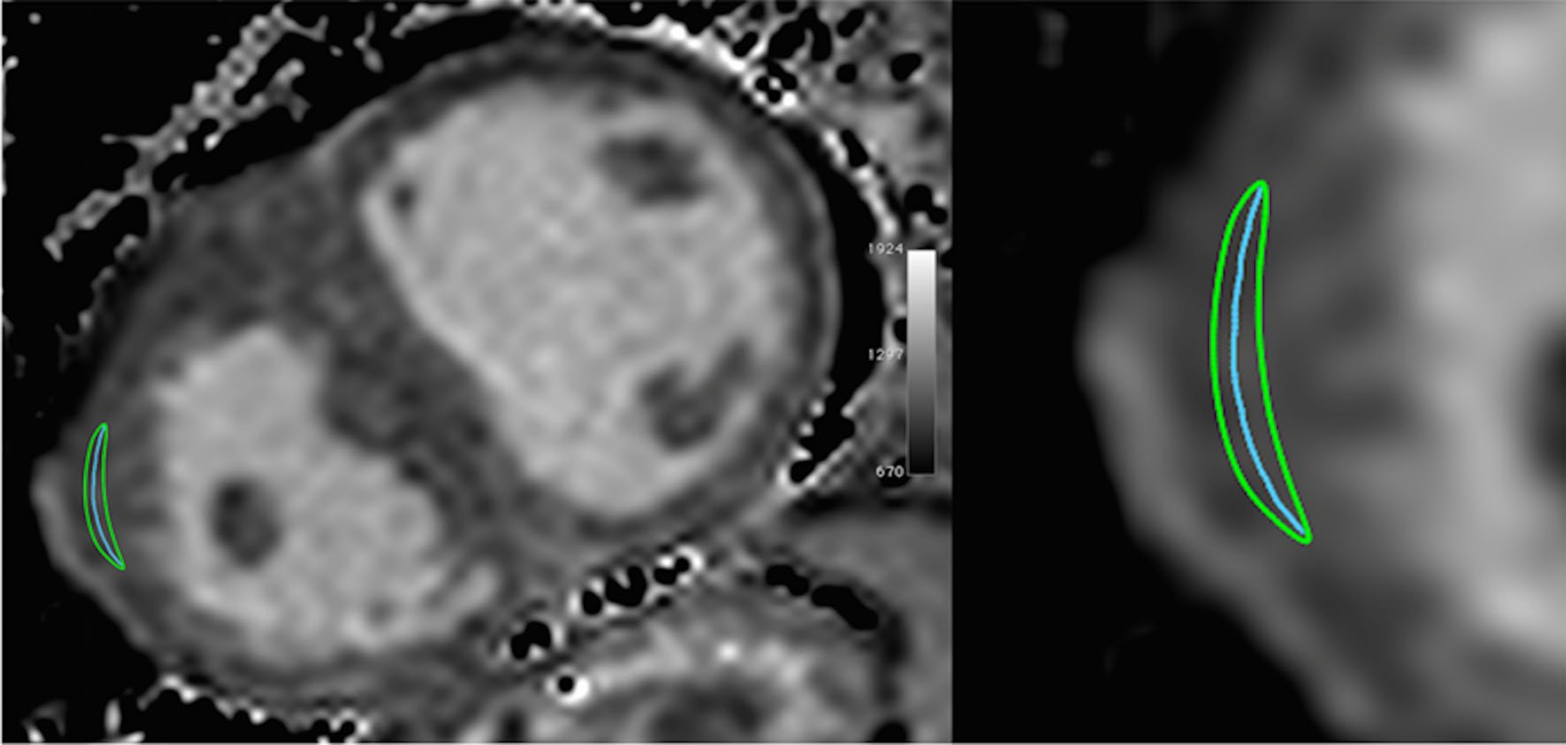
Example of T1 map in congenital heart disease. Representative native T1 map of a 47-year-old patient with palliated pulmonary atresia and ventricular septal defect showing region of interest (green) and corresponding line of interest (blue) in the right ventricle in short axis orientation. Images were created using OsiriX Lite (v.8.5, https://www.osirix-viewer.com/).

In addition to planimetric ECV analysis, RV ECV was determined with a novel centerline approach (Fig. 3). A curved “line of interest” (LOI) ≥ 10 mm in length was manually drawn in the center of the RV myocardium with a virtual pencil tool, leaving sufficient distance to the outer and inner border of the RV wall, but without the need for precise delineation between myocardium and neighboring blood or epicardial fat. The LOI approach uses the pixels crossed by the line to compute the mean T1 and the standard deviation (SD). Mean T1 values derived from the LOIs were used to measure RV ECV. For comparison between methods, conventional ROIs and corresponding LOIs were placed on identical myocardial regions in identical T1 maps. To account for the fact that the ROI method has not yet been validated for ECV measurements of the RV, LOI was compared with ROI as the “gold standard” for T1 analysis of the LV in a subset of ten patients with repaired TOF.

Hematocrit values were gained immediately before CMR to calculate ECV as follows^19,20^:

$${\text{ECV}}_{{{\text{myocardium}}}} { = }\left( {\text{1 - hematocrit}} \right){ } \times { }\left( {\frac{{\left[ {\frac{{1}}{{{\text{T1}}_{{\text{myocardium post}}} }}{ - }\frac{{1}}{{{\text{T1}}_{{\text{myocardium pre}}} }}} \right]}}{{\left[ {\frac{{1}}{{{\text{T1}}_{{\text{blood post}}} }}{ - }\frac{{1}}{{{\text{T1}}_{{\text{blood pre}}} }}} \right]}}} \right)$$

where post = T1 15 min after contrast, and pre = T1 before contrast (native).

Image analysis was undertaken by a single operator with 7 years of experience in CMR imaging in CHD. To test intraobserver variability of T1/ECV measurements, the same operator repeated the analysis in a subset of 10 randomly chosen CHD patients and 5 controls at least 2 weeks apart from the first time of measurements. For interobserver variability, a second observer with 5 years of experience in CMR, blinded to the clinical diagnosis and to other CMR results, performed image analysis in the same subset of patients.

### Statistical analysis

Normality was assessed with the Shapiro–Wilk test. Values are expressed as median (range), mean ± SD, or numbers (n) and percentages (%), as appropriate. Associations between continuous variables were assessed with Pearson´s or Spearman´s correlation coefficients (r). Bland–Altman analysis was applied to compare RV ECV from SAX and TRANS, and ECV from plane ROIs and corresponding LOIs within groups. Comparisons between patients and controls were performed with unpaired *t* test or Mann–Whitney U test. Intra- and interobserver variability were tested with Bland–Altman analysis and correlation coefficients. Statistical analyses were performed with SPSS version 24.0 (IBM Corp., Armonk, NY). A *p* value < 0.05 was considered statistically significant.

## Results

In total, 39 CHD patients and 17 controls were analyzed. Patient demographics and diagnoses are summarized in Table 2. In the control group, median age at CMR was 24 (23–28) years, and 9 (53%) were female. There were no significant differences between patients and controls regarding age (*p* = 0.310) and gender distribution (*p* = 0.640).

**Table 2:** Patient characteristics (n = 39).

**Age at CMR (years)**	28 (20–37)
**Female**/**Male**	18 (46)/21 (54)
**Diagnosis**	
Repaired TOF	12 (30.8)
Repaired PA + VSD	2 (5.1)
Repaired DORV	2 (5.1)
Dysplastic pulmonary valve	3 (7.7)
LPA hypoplasia	1 (2.6)
RPA stenosis	1 (2.6)
dTGA, ASO	3 (7.7)
Systemic right ventricle	8 (20.5)
dTGA, Senning	4 (10.3)
dTGA, Mustard	1 (2.6)
ccTGA	3 (7.7)
Univentricular heart	7 (17.9)
Fontan palliation	6 (15.4)
Cyanotic	1 (2.6)
**CMR data**	
RVEDVi (ml/m^2^)	111 (96–128)
RVESVi (ml/m^2^)	56 (46–67)
RVSVi (ml/m^2^)	50 (44–62)
RVEF (%)	50 (45–55)
Presence of RVH	23 (59)
Presence of LGE	23/35 (66)
PR fraction (%)*	29 (23–37)

Values are given as median (range) or n (%). *PR fraction determined in patients with repaired TOF, PA + VSD and DORV, and dysplastic pulmonary valve.

ASO = arterial switch operation; ccTGA = congenitally corrected transposition of the great arteries; CMR = cardiovascular magnetic resonance; DORV = double outlet right ventricle; dTGA = d-transposition of the great arteries; LGE = late gadolinium enhancement; LPA = left pulmonary artery; PA + VSD = pulmonary atresia with ventricular septal defect; PR = pulmonary regurgitation; RPA = right pulmonary artery; RVEDVi = indexed right ventricular end-diastolic volume; RVEF = right ventricular ejection fraction; RVESVi = indexed right ventricular end-systolic volume; RVH = right ventricular hypertrophy; RVSVi = indexed right ventricular stroke volume; TOF = tetralogy of Fallot.

### Short axis and transverse orientation

In CHD patients, correlation between the two orientations was strong for RV native T1 (0.84, *p* < 0.001) and ECV (r = 0.90, *p* < 0.001). Bland–Altman analysis showed no significant bias in native T1 or ECV between the different orientations. ECV could not be determined in SAX in 6/39 patients due to insufficient image quality (n = 2) or RV wall thickness (n = 4). In 14/39 patients, ECV analysis in TRANS was not possible because image quality was too low (n = 6; Fig. 1c) or RV walls were too thin (n = 7); in 1/14 patients the post contrast T1 map in TRANS was not available. In 2/6 patients with missing SAX values, ECV was alternatively measurable in TRANS.

In the control group, correlation between SAX and TRANS was strong for RV native T1 (r = 0.80, *p* = 0.030) and ECV (r = 0.89, *p* = 0.016), without significant bias in the Bland–Altman analysis. RV ECV measurements were not possible in SAX in 9/17 and in TRANS in 12/17 control subjects as RV walls were too thin to accurately delineate myocardium from extramyocardial tissues. In 1/9 without SAX values, ECV was available in TRANS.

RV ECV and LV ECV from SAX were significantly higher in CHD patients than in controls (RV: 0.31 ± 0.05 vs. 0.28 ± 0.02, *p* = 0.009; LV: 0.30 ± 0.05 vs. 0.26 ± 0.03, *p* = 0.001). Significant differences in native T1 of RV and LV between patients and controls were not observed (*p* = 0.475 and 0.354, respectively). In patients, native T1 and ECV of the RV in systemic vs. subpulmonary position were not significantly different (*p* = 0.945 and 0.279, respectively). RV native T1 and ECV did not correlate with RV end-diastolic volume index (r = 0.01, *p* = 0.965/r = 0.22, *p* = 0.216), end-systolic volume index (r = − 0.05, *p* = 0.763/r = 0.13, *p* = 0.475) or ejection fraction (r = − 0.05, *p* = 0.788/r = − 0.01, *p* = 0.941). An association with RV wall thickness per ROI in pixels was not seen (RV native T1/ECV: *p* = 0.292, and 0.734, respectively).

### Image quality and wall thickness

Image quality was high in patients and controls (mean score grade 1.3 and 1.1, respectively), and tended to be higher before than after contrast application (patients: 1.1 vs. 1.6; controls: 1.0 vs. 1.3). Maximum RV wall thickness per ROI was greater in CHD (1–2 pixels, mean score grade 2.2) than in controls (< 1 pixel, mean score grade 2.5; *p* < 0.001). The distribution of scores is shown in Table 3.

**Table 3:** Distribution of grades for image quality and right ventricular wall thickness.

	n	Image quality			RV wall thickness per ROI		
		SAX	TRANS	Total	SAX	TRANS	Total
**Controls**	17	1.2	1.1	1.1	2.5	2.6	2.5
**Patients total**	39	1.5	1.2	1.3	2.2	2.2	2.2
Repaired TOF	12	1.3	1.1	1.2	2.2	2.3	2.2
Repaired PA + VSD	2	1.3	1.0	1.1	2.0	2.0	2.0
Repaired DORV	2	1.5	1.0	1.3	2.5	2.8	2.6
Dysplastic PV	3	1.2	1.0	1.1	2.0	2.0	2.0
LPA hypoplasia	1	1.5	1.0	1.3	2.0	3.0	2.5
RPA stenosis	1	2.0	2.0	2.0	3.0	2.0	2.5
dTGA, ASO	3	1.3	1.0	1.2	2.0	2.0	2.0
Systemic RV	8	1.8	1.3	1.5	1.9	2.0	2.0
Univentricular heart	7	1.4	1.4	1.4	1.8	2.0	1.9
**Total**	56	1.4	1.2	1.2	2.4	2.4	2.4

Mean grades for image quality and maximum RV wall thickness per ROI are given.

Image quality: grade 1: no artifacts in RV, good contrast between blood and myocardium; grade 2: minor artifacts in RV, good/sufficient contrast between blood and myocardium; grade 3: major artifacts in RV and/or insufficient contrast between blood and myocardium.

Maximum RV wall thickness per ROI: grade 1: > 2 pixels; grade 2: 1–2 pixels; grade 3: < 1 pixel.

ASO = arterial switch operation; DORV = double outlet right ventricle; dTGA = d-transposition of the great arteries; LPA = left pulmonary artery; PA + VSD = pulmonary atresia with ventricular septal defect; PV = pulmonary valve; ROI = region of interest; RPA = right pulmonary artery; RV = right ventricle; SAX = short axis; TOF = tetralogy of Fallot; TRANS = transverse.

RV ECV could not be determined in 4/39 patients. Of those, one patient (univentricular heart, cyanosis) had insufficient image quality (score grade 3), and three patients (repaired TOF/double outlet right ventricle with pulmonary regurgitation (n = 1, respectively); right pulmonary artery stenosis) had minor RV wall thickness per ROI (< 1 pixel, score grade 3). In 8/17 controls, RV wall thickness was insufficient for RV ECV analysis.

### Region of interest and line of interest

**Figure 4:**
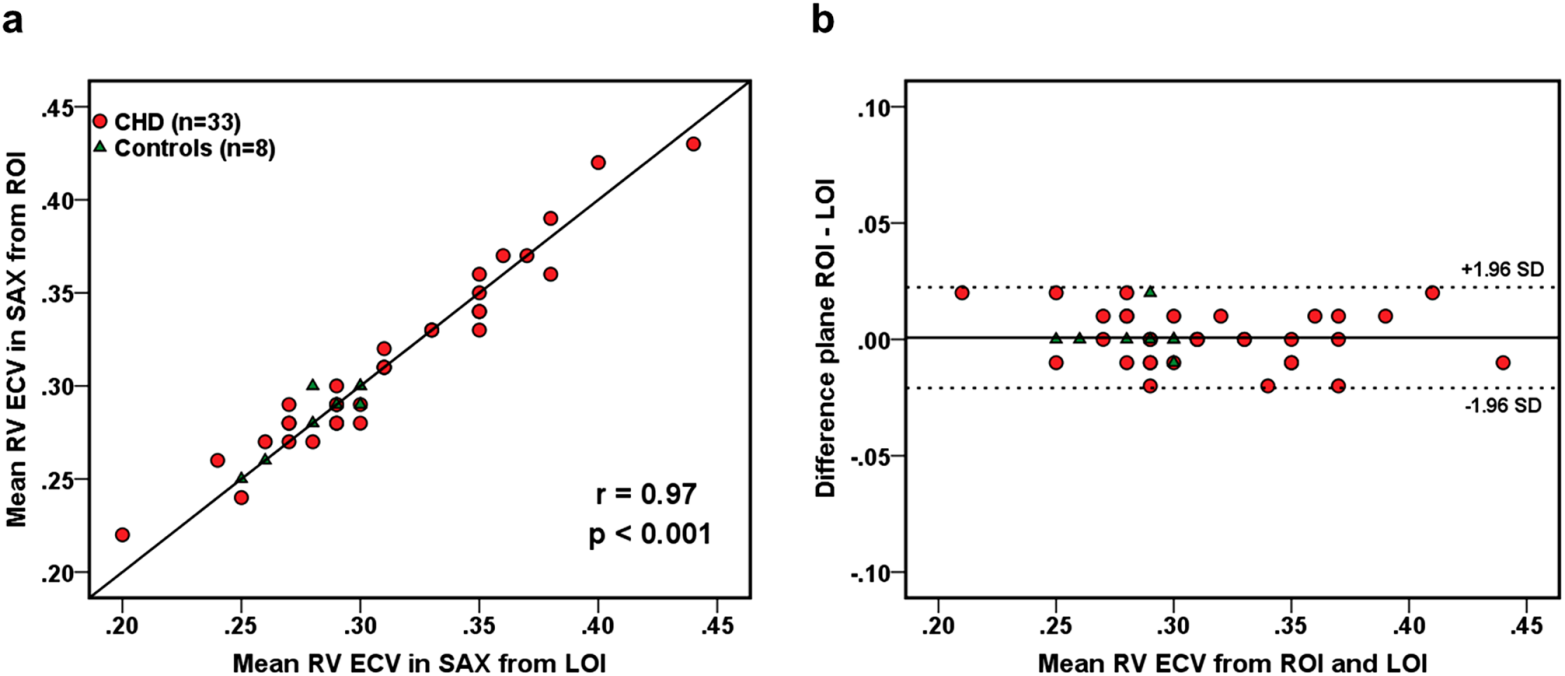
Extracellular volume—region of interest versus line of interest. Correlation (**a**) and Bland–Altman analysis (**b**) of right ventricular myocardial extracellular volume (RV ECV) from regions of interest (ROIs) and corresponding lines of interest (LOIs) in short axis orientation across healthy controls and patients with congenital heart disease.

RV native T1 from ROI agreed well with values from corresponding LOIs across health and CHD in SAX (r = 0.83, *p* < 0.001) and TRANS (r = 0.81, *p* < 0.001). With regard to RV ECV, correlation was very strong between values derived from ROI and LOI s in SAX (r = 0.97, *p* < 0.001; Fig. 4) and TRANS (r = 0.87, *p* < 0.001). Bland–Altman analysis revealed no significant bias between the two methods. An overview of RV native T1 and ECV values is given in Table 4.

**Table 4:** Native T1, post-contrast T1 and extracellular volume of the right ventricle from regions of interest and corresponding lines of interest in short axis and transverse orientation.

		Controls		Patients	
		SAX	TRANS	SAX	TRANS
**Native T1 (ms)**	**RV ROI**	1006 ± 49	981 ± 85	1018 ± 30	1007 ± 23
	**RV LOI**	1006 ± 42	984 ± 81	1014 ± 40	966 ± 183
	**Blood**	1551 ± 76	1541 ± 65	1492 ± 88	1450 ± 84
		(n = 10)	(n = 8)	(n = 35)	(n = 29)
**Post-contrast T1 (ms)**	**RV ROI**	617 ± 26	608 ± 23	596 ± 63	594 ± 68
	**RV LOI**	626 ± 35	612 ± 26	594 ± 62	599 ± 67
	**Blood**	538 ± 49	540 ± 37	531 ± 73	533 ± 70
		(n = 8)	(n = 7)	(n = 33)	(n = 25)
**ECV**	**RV ROI**	0.28 ± 0.02	0.28 ± 0.03	0.31 ± 0.05	0.31 ± 0.04
	**RV LOI**	0.28 ± 0.02	0.28 ± 0.03	0.31 ± 0.05	0.30 ± 0.04
		(n = 8)	(n = 7)	(n = 33)	(n = 25)
**Hematocrit**		0.40 ± 0.05		0.43 ± 0.05	

Values are given as mean ± standard deviation.

ECV = extracellular volume; LOI = line of interest; ROI = region of interest; RV = right ventricle; SAX = short axis; TRANS = transverse.

In a subset of patients with repaired TOF (n = 10), the LOI method was compared with ROI for T1 measurements of the LV. Agreement between ROI and LOI was moderate for native T1 (r = 0.66, *p* = 0.038) and strong for LV ECV (r = 0.83, *p* = 0.003).

### Reproducibility

There was good agreement of RV and LV ECV measurements within and between raters. Data for intra- and interobserver variability are summarized in Table 5.

**Table 5:** Intra-and interobserver variability for extracellular volume across healthy controls and patients with congenital heart disease.

	Intraobserver				Interobserver			
	Mean difference (SD)	95%-confidence interval	Limits of agreement	r (*p* value)	Mean difference (SD)	95%-confidence interval	Limits of agreement	r (*p* value )
**RV ECV (ROI)**	-0.004 (0.023)	-0.017–0.006	-0.049–0.041	0.80 (< 0.001)	-0.005 (0.025)	-0.022–0.011	-0.052–0.042	0.62 (0.041)
**RV ECV (LOI)**	-0.012 (0.028)	-0.027–0.003	-0.042–0.066	0.63 (0.012)	-0.005 (0.025)	-0.022–0.013	-0.052–0.039	0.76 (0.007)
**LV ECV**	-0.007 (0.011)	-0.015–0.000	-0.028–0.015	0.96 (< 0.001)	-0.002 (0.020)	-0.016–0–012	-0.044–0.041	0.79 (0.003)

ECV = extracellular volume; LOI = line of interest; r = Pearson´s correlation coefficient; ROI = region of interest; RV = right ventricle; SD = standard deviation.

## Discussion

In the present study, RV ECV could be determined in the majority of CHD subjects given that image quality was sufficient for differentiation between RV myocardium and blood pool, and maximum RV wall thickness per ROI was ≥ 1 pixel. Furthermore, LOI was introduced as a novel tool for RV ECV analysis.

Diffuse myocardial fibrosis has been associated with myocardial dysfunction and adverse clinical outcomes in different forms of CHD^6–11,21,22^. In patients with repaired TOF, increased ECV was associated with RV volume overload and arrhythmia. In that cohort, RV ECV was significantly elevated as compared to controls^8^. Similarly, we detected significantly higher RV and LV ECV in CHD patients than in healthy controls. Greater LV ECV in adults with TOF has also been described by Broberg et al. who found correlations with adverse clinical markers and outcomes^7^. Furthermore, reduced post-contrast RV and LV T1 values have been detected in children after TOF repair^13^. Consistent with our findings, these data may indicate increased diffuse myocardial fibrosis in both ventricles, in turn pointing to an adverse interventricular interaction in the context of cardiac remodeling. Previous work suggests that these interactions may contribute to heart failure, exercise intolerance, and arrhythmia in repaired TOF^7,8,22,23^.

In children post Fontan palliation with a systemic RV, increased markers of diffuse myocardial fibrosis negatively correlated with strain parameters from CMR feature tracking, suggesting an association with decreased myocardial contractility^10^. Plymen et al. measured higher septal ECV values in the systemic RV of patients with TGA after atrial direction compared with controls. ECV measurements of the RV free wall were abandoned due to partial voluming of the RV myocardium with blood^11^. Given the morphologic characteristics of the RV, delineation of its myocardium to accurately determine RV ECV is challenging. Combined measurements of myocardial and blood T1 will lead to altered myocardial ECV values with potential overestimation of the extent of diffuse myocardial fibrosis. In our study, plane ROIs were centered in the RV myocardium with particular care to prevent inclusion of neighboring blood or extramyocardial tissues. RV wall thickness of ≥ 1 pixel per ROI was identified to sufficiently minimize contamination of myocardial ROIs. The greater intra- and interobserver variability of RV ECV measurements in comparison with LV ECV may however mirror the potential difficulties of accurate RV wall demarcation excluding adjacent blood or extramyocardial tissues. A different approach for RV ECV quantification has been described by Mehta et al. using accelerated and navigator-gated Look-Locker imaging for cardiac T1 estimation (ANGIE) in patients with pulmonary hypertension. In that cohort, RV ECV was significantly higher than in healthy volunteers and patients with heart failure and reduced LV ejection fraction but without pulmonary hypertension^24^.

For RV volumetric analysis, cine imaging in transverse (axial) plane has been recommended, as it allows for better recognition of the anatomic landmarks of the RV and improved visual assessment of RV longitudinal contractility, in turn leading to more reproducible measurements of RV volumes^25^. In our study, RV ECV from SAX and TRANS correlated strongly in patients and controls. However, the RV myocardium tended to be more clearly delineated in SAX, resulting in a higher number of measurable T1 data sets, and thus suggesting a more reliable measurability of RV ECV. We would therefore recommend T1 maps in SAX for RV analysis. In individual subjects, T1 images in TRANS may additionally be acquired as a potentially useful alternative if ROIs cannot accurately be placed in the RV myocardium in SAX.

RV ECV from plane ROIs agreed well with values from corresponding LOIs. To account for the fact that the ROI method has not yet been validated for ECV measurements of the RV, LOI was compared with ROI as the “gold standard” for T1 analysis of the LV. In line with the RV, correlation between LV ECV values from ROI and LOI was strong. Instead of contouring the myocardium, the centerline approach targets the ventricular wall directly in its center without the need for differentiation between myocardial and blood pixels. The application of this custom-made tool facilitates a quick and simple evaluation of myocardial RV ECV, and thus may support the integration of ECV measurements into clinical CMR workflows. Furthermore, this approach may in the future also prove to be useful for the assessment of other thin cardiac structures, e.g. atrial walls or thin LV myocardium in dilated cardiomyopathy.

In addition to ECV, native T1 is a commonly applied T1 mapping metric to evaluate myocardial fibrosis. In patients with cardiomyopathy, native T1 provided the greatest distinction between healthy and diseased myocardium compared to post-contrast T1 and ECV^26^. In our study, both native T1 and ECV correlated similarly between T1 map orientations and measurement methods, while significant differences between patients and controls were only seen with regard to ECV. Especially in patients with CHD and the need for serial CMR examinations, a non-contrast approach would be favorable. Furthermore, T1 mapping of the RV can particularly be challenging after contrast, as underlined by the lower image quality of post-contrast T1 maps seen in this study. Whether native T1 is a reliable marker of myocardial disease in CHD remains to be proven.

Given the key role of diffuse myocardial fibrosis within the multi-causal development of heart failure and the possible efficacy of antifibrotic treatment^27,28^, further research is required to assess the potential of non-invasive T1 mapping parameters within the clinical management of CHD.

The present study has several limitations. The patient cohort is relatively small and heterogeneous, making it difficult to draw specific conclusions relating to the higher ECV but similar native T1 values when compared to the healthy controls. Given the age of the study population, results should be confirmed in pediatric cohorts with younger CHD patients taking into account potential spatial and temporal resolution issues. Histological validation of ECV was not performed since biopsy and/or surgery was not clinically indicated in any of the enrolled patients. Previous studies have shown good agreement between LV ECV from T1 mapping and collagen volume fraction from myocardial biopsy in aortic stenosis^12,29^, hypertrophic^12^, and dilated cardiomyopathy^30^, while RV ECV yet needs to be validated. Although particular care was taken when drawing a ROI into the RV myocardium at a minimum wall thickness of > 1 pixel, partial voluming with blood or fat due the thin myocardium and trabeculation of the RV could potentially have led to an overestimation of ECV, in turn limiting the accuracy of RV ECV measurements. It has been shown that LV ECV values are reproducible across CMR scans^31^. Since serial CMR examinations were not performed in the present study, test–retest reliability of RV ECV measurements could not be analyzed.

In conclusion, our study demonstrated that non-invasive evaluation of RV ECV by CMR T1 mapping is feasible in CHD provided that image quality allows for sufficient distinction between myocardium and blood, and that RV wall thickness per ROI is ≥ 1 pixel. Furthermore, the application of a manually drawn LOI centered in the RV myocardium simplifies RV ECV analysis. Future studies are necessary to further explore the potential role of myocardial ECV as a non-invasive marker for prognosis, risk stratification, and monitoring of disease and therapy in CHD.

## Data Availability

The datasets generated during and/or analysed during the current study are available from the corresponding author on reasonable request.
